# Awareness of and Hypothetical Willingness to Use Long-Acting Injectable Cabotegravir and Associated Factors Among Oral Pre-Exposure Prophylaxis Users Who Obtained the Medicine Online in China: Web-Based Cross-Sectional Study

**DOI:** 10.2196/96586

**Published:** 2026-10-28

**Authors:** Qingyu Li, Jingtao Zhou, Siwen Huang, Yuhang Zhang, Jiayin Zheng, Chi Ruan, Huachun Zou, Xianlong Ren, Jie Xu, Houlin Tang, Yutong Xu, Zhiyi Zhao, Hui Xue, Feng Cheng, Sitong Luo

**Affiliations:** 1 Vanke School of Public Health, Tsinghua University, 30 Shuangqing Rd, Haidian District, Beijing, 100084, China, +86 010 62791154; 2 School of Public Health, Fudan University, Shanghai, China; 3 Department of AIDS/STD Control and Prevention, Beijing Center for Disease Control and Prevention, Beijing, China; 4 National Center for AIDS/STD Control and Prevention, Chinese Center for Disease Control and Prevention, Beijing, China; 5 HeHealth, Beijing, China; 6 Institute for Healthy China, Tsinghua University, Beijing, China

**Keywords:** long-acting injectable cabotegravir, pre-exposure prophylaxis, awareness, willingness, online platforms, China

## Abstract

**Background:**

Oral pre-exposure prophylaxis (PrEP) is highly effective for HIV prevention, but its real-world impact depends on sustained adherence. Long-acting injectable cabotegravir (CAB-LA) may reduce the burden of daily or event-driven pill use. In China, many oral PrEP users obtain medication online, but little is known about their prior CAB-LA awareness or willingness to consider it.

**Objective:**

This study assessed prior CAB-LA awareness, hypothetical willingness to use CAB-LA, reported barriers and facilitators, and associated factors among oral PrEP users in China obtaining PrEP online.

**Methods:**

This cross-sectional study analyzed baseline data from the Real-world E-consumer Cohort of Oral PrEP (RECOPE), a prospective cohort in China. Participants completed a self-administered web-based questionnaire assessing prior CAB-LA awareness, hypothetical willingness after standardized CAB-LA information, sociodemographic characteristics, recent sexual behaviors and sexually transmitted infection (STI) diagnosis, and oral PrEP-related characteristics. Modified Poisson regression with robust SEs estimated prevalence ratios (PRs) and adjusted prevalence ratios (aPRs) with 95% CIs. Primary regression analyses were restricted to sexually active participants.

**Results:**

Among 621 oral PrEP users, the mean age was 29.9 (SD 6.4) years; all were assigned male sex at birth, and 608 (97.9%) identified as men. Overall, 216 of 621 (34.8%, 95% CI 31.1%‐38.6%) participants reported prior CAB-LA awareness. After receiving standardized information, 428 of 621 (68.9%, 95% CI 65.2%‐72.4%) participants expressed hypothetical willingness to use CAB-LA if it became available in China. Convenience was the most frequently reported facilitator among willing participants (395/428, 92.3%), whereas high cost was the most frequently reported barrier among those who did not express willingness (123/193, 63.7%). In adjusted analyses, event-driven PrEP users had a lower prevalence of prior CAB-LA awareness than daily PrEP users (aPR 0.66, 95% CI 0.50‐0.86), whereas participants who had switched oral PrEP regimens in the past 3 months had higher prior awareness (aPR 1.34, 95% CI 1.07‐1.68). Hypothetical willingness was more prevalent among participants reporting an STI diagnosis in the past 3 months (aPR 1.22, 95% CI 1.08‐1.39) and chemsex (aPR 1.13, 95% CI 1.01‐1.26), whereas higher PrEP-related stigma was associated with slightly lower willingness (aPR 0.99 per 1-point increase, 95% CI 0.98‐0.99).

**Conclusions:**

Among oral PrEP users in China obtaining PrEP online, prior CAB-LA awareness was limited, whereas hypothetical willingness was common after standardized information was provided. Prior awareness differed by oral PrEP regimen and recent regimen switching, whereas willingness was associated with recent STI diagnosis, chemsex, and PrEP-related stigma. Convenience was a major appeal, while cost remained an important barrier. These findings support improving access to balanced PrEP information and addressing practical barriers as long-acting PrEP becomes more widely available.

## Introduction

Over the past decade, pre-exposure prophylaxis (PrEP) has become a proven and highly effective strategy for HIV prevention. Randomized controlled trials have demonstrated that oral tenofovir- or emtricitabine-based PrEP substantially reduces HIV acquisition among key populations, including men who have sex with men (MSM) and sex workers [1,2]. Although oral PrEP is highly effective, its real-world effectiveness depends heavily on sustained adherence. Suboptimal adherence has been associated with reduced protective efficacy and diminished population-level impact [2]. The adherence challenge is especially pronounced among event-driven users who account for the majority of real-world PrEP users in some countries such as China, as they have to decide on dose timing in relation to sexual activity repeatedly following the “2-1-1” protocol [3]. Adherence to oral PrEP is often undermined by practical and psychosocial barriers, such as difficulties in remembering doses, low perceived HIV risk, and PrEP-related stigma [4]. In China, these challenges are further compounded by structural constraints, including out-of-pocket costs, limited provider engagement, and persistent cultural stigma toward HIV and same-sex behaviors [5-7].

In parallel with the expansion of PrEP use, telemedicine-based and online models of PrEP delivery have emerged in several countries, including the United States, Brazil, and China, partly because they offer greater convenience and privacy than conventional clinic-based pathways [8,9]. In these models, users may complete web-based eligibility assessments, receive remote consultation, and obtain medication through online platforms. In China, where eHealth commerce platforms have become the primary channel for PrEP purchasing, nearly 70% of real-world PrEP users obtain PrEP online, according to the Chinese Center for Disease Control and Prevention (CDC) [7]. Although such models may improve convenience and privacy, they may also provide less direct counseling and fewer opportunities for face-to-face support, thereby shaping how users access PrEP-related information and make prevention decisions.

Long-acting injectable cabotegravir (CAB-LA), an integrase strand transfer inhibitor, offers an alternative prevention option that may reduce some adherence challenges associated with oral regimens [10]. After 2 initiation injections given 1 month apart, CAB-LA is administered every 2 months for as long as HIV exposure risk persists. In the HPTN 083 and HPTN 084 trials, CAB-LA showed higher efficacy than daily oral PrEP in preventing HIV infection among populations at substantial risk [10,11]. CAB-LA has been recommended by the World Health Organization as an additional HIV prevention option [12]. At the time of data collection, CAB-LA had not yet been approved for HIV prevention in China; the PrEP indication was subsequently approved in May 2024 and incorporated into the 2024 Chinese HIV guidelines [13]. However, the transition from clinical efficacy and regulatory approval to routine use in real-world settings is not automatic. The successful implementation of CAB-LA depends not only on its pharmacological advantages but also on whether potential users are aware of this option and are willing to consider it as an alternative prevention strategy during the early implementation phase.

Existing studies in China have primarily examined CAB-LA perceptions among general MSM populations [14-17]. Although these studies provide useful early evidence, they may not reflect the views of individuals who are already using PrEP and making decisions about prevention modality. For current oral PrEP users, CAB-LA is not simply a hypothetical option but a possible alternative to an existing strategy [18,19]. Nevertheless, most existing evidence comes from clinical or demonstration trials, where medication is provided free of charge and participants receive intensive counseling and follow-up. Such decision-making contexts differ from those of real-world oral PrEP users in China, many of whom obtain PrEP through online platforms and must consider costs, service access, and long-term commitment to injectable prevention. Consequently, awareness of and willingness to use CAB-LA in this population remain insufficiently understood. Therefore, this study aimed to examine the awareness of CAB-LA, willingness to use it, and associated factors among real-world oral PrEP users in China who obtain PrEP through online platforms.

## Methods

### Study Design and Participants

This was a cross-sectional analysis of baseline data from the Real-world E-consumer Cohort of Oral PrEP (RECOPE), a prospective cohort of oral PrEP users in China obtaining PrEP online. Recruitment and baseline assessment for RECOPE were conducted from December 6, 2023, to January 4, 2024, followed by assessments at 1, 3, and 6 months. The cohort design and adherence-related findings have been reported previously [7]. The present analysis focused on real-world oral PrEP users obtaining PrEP online, defined as participants who had accessed oral PrEP through the online PrEP service platform and had initiated oral PrEP before the baseline assessment. This study followed the Checklist for Reporting Results of Internet E-Surveys (CHERRIES; Checklist 1).

Participants were recruited via HeHealth (formerly known as BluedHealth), the leading internet-based PrEP distribution platform in China and Asia. Eligible participants were required to be aged 18 years or older, to have purchased oral PrEP online within the preceding 1 to 3 months and completed the platform’s routine PrEP eligibility assessment, to plan to continue PrEP use during the following 6 months, and to provide electronic informed consent. The platform’s routine eligibility assessment included self-reported information on HIV exposure risk, recent HIV testing, and potential contraindications to PrEP, which was reviewed remotely by a physician before an electronic prescription was issued. During study screening, individuals were excluded if they were unable to read and understand the Chinese-language study materials, respond to the screening questions, or communicate sufficiently with the research team to complete the study procedures. A total of 657 participants completed the RECOPE baseline survey. After excluding 36 participants who had purchased but had not initiated oral PrEP, 621 real-world oral PrEP users obtaining PrEP online were included in the present analysis.

### Recruitment and Web-Based Survey Procedures

During the recruitment period, HeHealth staff sent an internal study invitation to all platform users who had purchased oral PrEP within the past 1 to 3 months. Users who expressed interest were initially screened for eligibility and, if potentially eligible, were referred to the research team through WeChat for further screening, electronic informed consent, and study enrollment.

After enrollment, each participant was assigned a unique study ID, which served as the passcode for accessing the web-based questionnaire hosted on Wenjuanxing [20]. The survey was not openly accessible to the public, and each study ID permitted only 1 questionnaire submission. Duplicate or unsolicited submissions were therefore primarily minimized through this restricted ID-based access mechanism. No IP address– or device-based restrictions were applied, and no separate automated bot detection system was used. All applicable questionnaire items presented to participants were mandatory. The questionnaire was self-administered and required approximately 5 to 10 minutes to complete. Three attention check items were embedded in the questionnaire, and participants were required to answer at least 2 correctly for their responses to be considered valid.

HeHealth was responsible for identifying and initially contacting potentially eligible users through the platform but did not have access to individual questionnaire responses. Further eligibility screening, informed consent, questionnaire administration, data management, and statistical analysis were conducted by the research team. No formal patient or public involvement was undertaken in the study design, analysis, or interpretation. Before formal data collection, the questionnaire was pilot-tested among 8 oral PrEP users, and their feedback was used to refine unclear wording before formal administration.

### Ethical Considerations

The study was approved by the Institutional Review Board of Tsinghua University (approval 20230195). Participation was voluntary, and electronic informed consent was obtained before enrollment. Participants reviewed an electronic informed consent form sent by the research team through WeChat, which described the study objectives, procedures, potential risks and benefits, confidentiality protections, and the voluntary nature of participation. Participants indicated their consent by clicking the agreement button before proceeding with the study. This procedure was used instead of obtaining a handwritten signature to reduce the collection of personally identifying information.

Participant confidentiality was protected by using study IDs rather than names or other direct personal identifiers in the questionnaire dataset. Individual questionnaire responses were accessible only to the research team and were not available to HeHealth. Participants received a HeHealth voucher valued at CNY 50 (CNY 1=US $0.14 as of January 4, 2024) after completing each valid questionnaire.

### Measures

#### Awareness of CAB-LA

The participants were first asked, “Have you ever heard of long-acting injectable cabotegravir (CAB-LA) for HIV prevention?”, with response options of “yes,” “no,” and “unsure.” Participants responding “yes” were classified as aware of CAB-LA, whereas those responding “no” or “unsure” were classified as not aware.

#### Hypothetical Willingness to Use CAB-LA

Before willingness was assessed, all participants were provided with the same standardized description of CAB-LA. The description summarized CAB-LA administration and dosing schedule, efficacy, common adverse effects, HIV testing and resistance-related considerations, and an estimated annual cost of approximately CNY 50,000. Hypothetical willingness to use CAB-LA was then assessed with the following item: “If CAB-LA becomes available for HIV prevention in China, would you be willing to use it?” Responses were recorded on a 5-point Likert scale ranging from 1 (“definitely unwilling”) to 5 (“strongly willing”). Consistent with prior research, responses of 4 or 5 were classified as willing, whereas responses of 1 to 3 were classified as not expressing willingness [14]. CAB-LA had not yet been approved for HIV prevention in China at the time of the baseline survey; therefore, the willingness measure reflected participants’ hypothetical preferences before domestic regulatory approval and commercial availability (Multimedia Appendix 1).

Participants were subsequently asked to select all applicable reasons for their willingness (eg, “it is more convenient” and “it provides longer-term protection against HIV”) or unwillingness (eg, “It is expensive” and “It may cause discomfort or pain from injections”) to use CAB-LA.

### Same-Sex Sexual Behaviors and Sexually Transmitted Infection Diagnoses

Same-sex sexual behaviors in the past 3 months were assessed, including the number of same-sex sexual partners, sexual role, condom use, chemsex, and commercial sex. Sexual role was categorized as insertive, receptive, or versatile. For analysis, a binary receptive anal sex variable was derived from sexual role, with participants reporting receptive or versatile roles classified as having receptive anal sex and those reporting an exclusively insertive role classified as not having receptive anal sex. Condom use frequency was assessed as never, occasionally, sometimes, often, or always and was further categorized as inconsistent use (never, occasionally, or sometimes) or consistent use (often or always). Chemsex was assessed by asking participants whether they had used any predefined psychoactive substances during same-sex sexual activity, including hallucinogens, inhalants, cannabis-related substances, and amphetamine-type stimulants; participants reporting the use of at least 1 listed substance were classified as having engaged in chemsex. Commercial sex was assessed based on whether participants had paid for sex or received payment for sex; reporting either behavior was classified as having engaged in commercial sex. Participants were also asked whether they had been diagnosed with syphilis, gonorrhea, genital warts, or genital herpes in the past 3 months.

### Oral PrEP-Related Characteristics

Participants reported their current oral PrEP regimen (daily or event-driven) and whether they had switched regimens during the past 3 months, with the direction of switching also recorded. PrEP adherence was assessed using regimen-specific criteria. Among sexually active event-driven PrEP users, optimal adherence was defined as PrEP use for every sexual encounter with correct adherence to the recommended 2-1-1 dosing schedule. Among daily PrEP users, optimal adherence was defined as reporting no missed or incorrectly timed doses in the past month. Participants who did not meet these stringent criteria were classified as not meeting the study-defined optimal adherence criteria. Knowledge of oral PrEP medication instructions was assessed using a regimen-specific, criterion-referenced assessment based on the recommended dosing instructions for event-driven and daily PrEP use. The event-driven use assessment included 5 response options reflecting the 2-1-1 dosing instructions, whereas the daily use assessment included 6 response options reflecting daily PrEP use instructions. Participants could select multiple options and were classified as having correct medication instruction knowledge only if they selected all correct options and no incorrect options; any omitted correct option or selected incorrect option was classified as incorrect. To support content validity and clinical accuracy, the regimen-specific items and response options were reviewed before formal administration by CDC experts with expertise in HIV prevention and PrEP. Participants also reported their approximate monthly expenditure on oral PrEP in Chinese yuan (CNY).

### PrEP-Related Stigma

PrEP-related stigma was assessed using an 11-item Chinese-language version of the HIV PrEP Stigma Scale [21]. The original English items were translated into Chinese by bilingual members of the research team and subsequently back-translated into English to assess semantic consistency with the original scale. The translated items were included in the pilot testing of the complete questionnaire among 8 oral PrEP users, and wording that was considered unclear or difficult to understand was refined before formal administration. Each item was rated on a 5-point Likert scale from 1 (strongly disagree) to 5 (strongly agree), with higher total scores indicating greater PrEP-related stigma. Internal consistency was good in the present sample (Cronbach α=0.81).

### Sociodemographic Characteristics

Baseline demographic information included age, ethnicity, sex assigned at birth, gender identity, employment status, education level, marital status, and monthly income. Gender identity was assessed as man, woman, transgender man, transgender woman, nonbinary, or another self-identified gender and was collapsed into man vs others for descriptive reporting because of the small numbers in the non-man categories. Employment status was categorized as employed vs student or unemployed; marital status as married or living with a partner vs unmarried, divorced, separated, or widowed; education as college or above vs high school or below; and monthly income as >CNY 7000 vs ≤CNY 7000.

### Statistical Analysis

Demographic characteristics were summarized using means and SDs for continuous variables and frequencies and percentages for categorical variables.

The prevalence of CAB-LA awareness and hypothetical willingness was reported with 95% CIs calculated using the Wilson method.

Given the cross-sectional design and the relatively common binary outcomes, modified Poisson regression with robust SEs was used to estimate prevalence ratios (PRs) and adjusted prevalence ratios (aPRs) with 95% CIs. Univariable models were first fitted to estimate crude PRs. Candidate covariates for the multivariable models were selected based on previous literature and their conceptual relevance to CAB-LA awareness or willingness among variables collected in the RECOPE baseline assessment. All selected covariates were entered simultaneously into the multivariable models irrespective of their statistical significance in univariable analyses. Prior awareness of CAB-LA was additionally included in the willingness model to account for pre-existing familiarity with CAB-LA before participants received the standardized information used for the willingness assessment.

Because sexual behavior measures were conditional on reporting sexual activity in the past 3 months, the primary regression analyses were restricted to sexually active participants (n=546). Oral PrEP adherence was defined using regimen-specific criteria and was included together with oral PrEP regimen in the pooled models. Given differences in adherence assessment between daily and event-driven PrEP, sensitivity analyses were conducted after excluding adherence from the models. For the willingness outcome, an additional sensitivity analysis excluded prior CAB-LA awareness. To assess whether restriction to sexually active participants influenced the findings, additional modified Poisson models were fitted using the full analytic sample (N=621), excluding sexual behavior variables and adherence that were structurally inapplicable to some participants and including recent sexual activity as a covariate.

Multicollinearity was assessed using variance inflation factors (VIFs). All applicable questionnaire items presented to participants were mandatory; unavailable values for conditional measures arose from prespecified skip patterns or structural inapplicability rather than item nonresponse, and no missing data imputation was performed. No separate a priori sample size calculation was conducted for this secondary analysis because the available sample was fixed by the RECOPE baseline cohort; all eligible baseline participants who had initiated oral PrEP were included in the analytic sample. All tests were 2-sided, with *P*<.05 considered statistically significant. Analyses were performed using R software (version 4.4.1; R Foundation for Statistical Computing).

## Results

### Participant Characteristics

A total of 621 oral PrEP users were included in the analysis. Of 877 users invited, 146 did not respond to the study invitation, 33 declined to participate after further communication, and 18 did not plan to continue PrEP use during the following 6 months. The remaining 680 eligible users were sent the baseline questionnaire, of whom 657 completed it, corresponding to a baseline questionnaire completion rate of 96.6% (657/680; Figure 1). The mean age was 29.9 (SD 6.4) years; of the 621 participants, 582 (93.7%) were of Han ethnicity, and 588 (94.7%) had completed college or higher education. All participants were assigned male sex at birth; of the 621 participants, 608 (97.9%) identified as men, and 13 (2.1%) reported other gender identities. More than half (363/621, 58.5%) reported a monthly income above CNY 7000.

**Figure 1.**
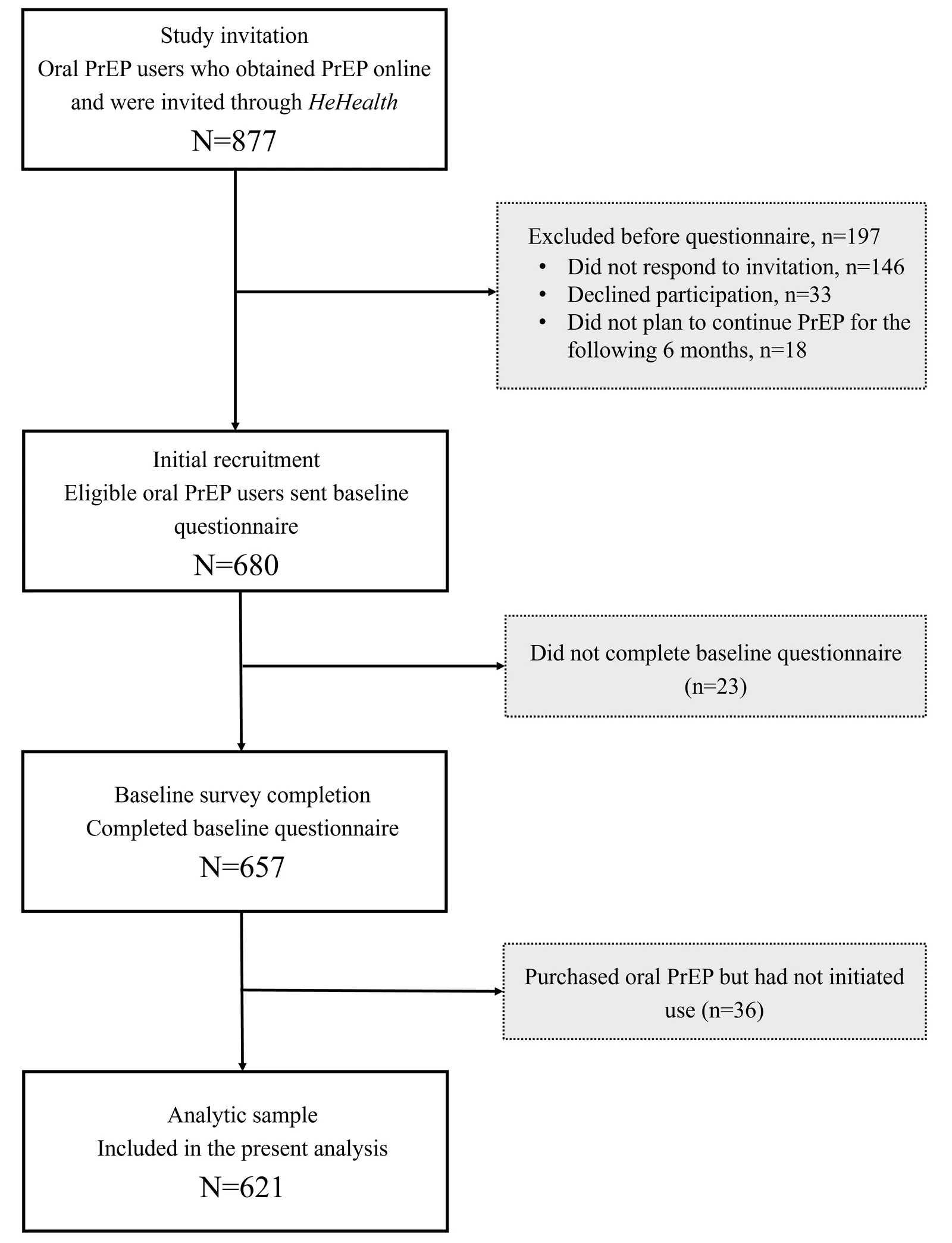
Flowchart of participant recruitment and inclusion in the present analysis. Participants were invited through HeHealth, an online oral PrEP service platform. Of 877 invited users, 680 met the eligibility criteria and were sent the baseline questionnaire, 657 completed the baseline survey, and 621 current oral PrEP users were ultimately included in the present analysis after excluding participants who had purchased but not initiated PrEP. PrEP: pre-exposure prophylaxis.

Most participants were using event-driven PrEP (529/621, 85.2%), while 92 of 621 (14.8%) participants were using daily PrEP. The median monthly expenditure on oral PrEP was CNY 300 (IQR 200‐300). Among the 550 participants for whom adherence was evaluable, 254 (46.2%) met the study-defined criteria for optimal adherence. Overall, 210 of 621 (33.8%) participants reported switching PrEP regimens during the past 3 months; 77 reported switching from event-driven to daily PrEP use, 85 from daily to event-driven PrEP use, and 48 reported multiple switches. In total, 62 of 621 (10%) participants reported a sexually transmitted infection (STI) diagnosis in the past 3 months. Among the 546 participants reporting sexual activity in the past 3 months, 237 (43.4%) reported chemsex, 210 (38.5%) reported inconsistent condom use, 445 (81.5%) reported more than 1 same-sex sexual partner, 256 (46.9%) reported receptive anal sex, and 48 (8.8%) reported commercial sex (Table 1).

**Table 1.** Characteristics of participants in this study (N=621)^a^.

Characteristic	Overall value
Background information	
Age (years), mean (SD)	29.9 (6.4)
18‐29, n (%)	337 (54.3)
30‐39, n (%)	235 (37.8)
≥40, n (%)	49 (7.9)
Sex assigned at birth, n (%)	
Male	621 (100)
Female	0 (0)
Ethnicity, n (%)	
Han	582 (93.7)
Others	39 (6.3)
Employment, n (%)	
Students/unemployed	123 (19.8)
Employed	498 (80.2)
Education level, n (%)	
High school and below	33 (5.3)
College and above	588 (94.7)
Marital status, n (%)	
Married or living with a partner	88 (14.2)
Unmarried/divorced/separated/widowed	533 (85.8)
Monthly income (CNY)^b^, n (%)	
≤7000	258 (41.5)
>7000	363 (58.5)
Gender identity, n (%)	
Man	608 (97.9)
Others	13 (2.1)
Same-sex sexual behaviors and recent STI^c^ diagnosis	
Chemsex, n (%)^d^	
Yes	237 (43.4)
No	309 (56.6)
Condom use, n (%)^d^	
Inconsistent	210 (38.5)
Consistent	336 (61.5)
Number of same-sex sexual partners, n (%)^d^	
1	101 (18.5)
>1	445 (81.5)
Receptive anal sex, n (%)^d^	
Yes	256 (46.9)
No	290 (53.1)
Commercial sex, n (%)^d^	
Yes	48 (8.8)
No	498 (91.2)
STI diagnosis in the past 3 months, n (%)	
Yes	62 (10)
No	559 (90)
PrEP^e^-related variables	
Oral PrEP regimen, n (%)	
Event-driven	529 (85.2)
Daily	92 (14.8)
Monthly expenditure on oral PrEP (CNY), median (IQR)	300 (200‐300)
Oral PrEP medication-instruction knowledge^f^, n (%)	
Correct	432 (69.6)
Incorrect	189 (30.4)
Study-defined optimal oral PrEP adherence^g^, n (%)	
Met criteria	254 (46.2)
Did not meet criteria	296 (53.8)
Oral PrEP regimen switch, n (%)	
Yes	210 (33.8)
No	411 (66.2)
PrEP-related stigma score, mean (SD)	25.0 (6.9)

a Values are presented as n (%) for categorical variables. Percentages were calculated using the number of participants for whom each measure was applicable as the denominator.

b CNY: Chinese yuan; CNY 1=US $0.14 as of January 4, 2024.

c STI: sexually transmitted infection.

d Sexual-behavior measures were assessed among participants reporting sexual activity in the past 3 months (n=546).

e PrEP: pre-exposure prophylaxis.

f Correct oral PrEP medication-instruction knowledge required correct responses to all regimen-specific items.

g Oral PrEP adherence was assessed using regimen-specific criteria and was evaluable for 550 participants; it was structurally inapplicable to 71 event-driven PrEP users reporting no sexual activity in the past 3 months.

### Awareness of and Hypothetical Willingness to Use CAB-LA

Among the 621 participants, 216 (34.8%, 95% CI 31.1%‐38.6%) reported prior awareness of CAB-LA. Prior awareness was reported by 47 of 92 (51.1%) daily PrEP users and 169 of 529 (31.9%) event-driven PrEP users. After receiving standardized information about CAB-LA, 428 of 621 (68.9%, 95% CI 65.2%‐72.4%) participants expressed hypothetical willingness to use CAB-LA if it became available in China (Figure 2). Among participants with prior awareness of CAB-LA, 156 of 216 (72.2%) expressed willingness, compared with 272 of 405 (67.2%) among those without prior awareness.

**Figure 2.**
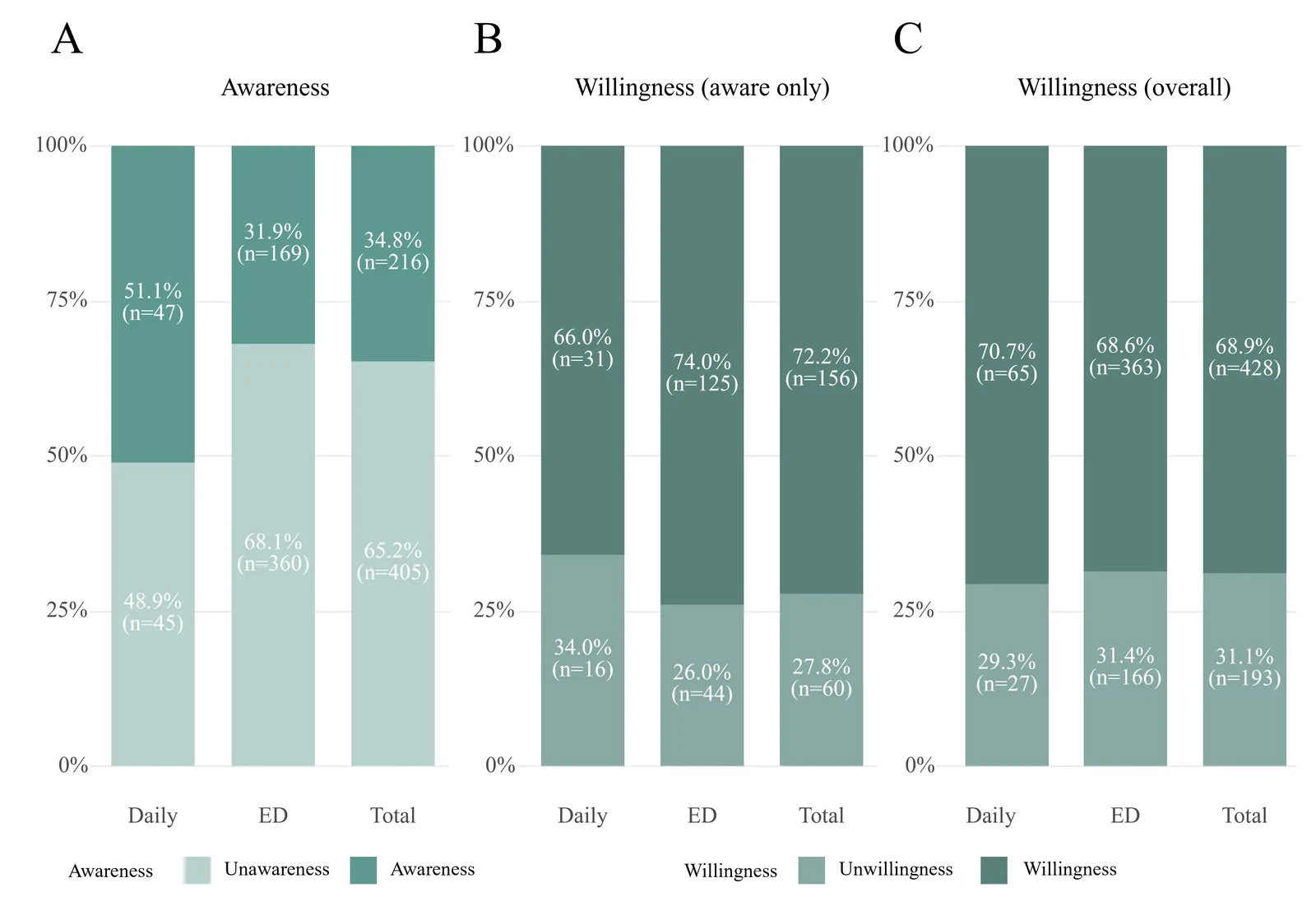
Prior awareness of and hypothetical willingness to use CAB-LA by oral PrEP regimen. Panel A shows prior awareness of CAB-LA among daily PrEP users (n=92), ED PrEP users (n=529), and the overall sample (N=621). Panel B shows hypothetical willingness to use CAB-LA among participants who reported prior awareness of CAB-LA (daily PrEP: n=47; ED PrEP: n=169; total: N=216). Panel C shows hypothetical willingness among all participants after they received standardized information about CAB-LA (daily PrEP: n=92; ED PrEP: n=529; total: N=621). Percentages were calculated within each regimen-specific or overall subgroup. CAB-LA: long-acting injectable cabotegravir; ED: event-driven; PrEP: pre-exposure prophylaxis.

Among the 428 participants who expressed hypothetical willingness to use CAB-LA, the most frequently reported facilitators were greater convenience (n=395, 92.3%), long-term prevention (n=343, 80.1%), and greater privacy (n=221, 51.6%). Among the 193 participants who did not express willingness, the most frequently reported barriers were high cost (n=123, 63.7%), injection-related pain (n=117, 60.6%), and concerns about adverse effects (n=99, 51.3%) (Figure 3).

**Figure 3.**
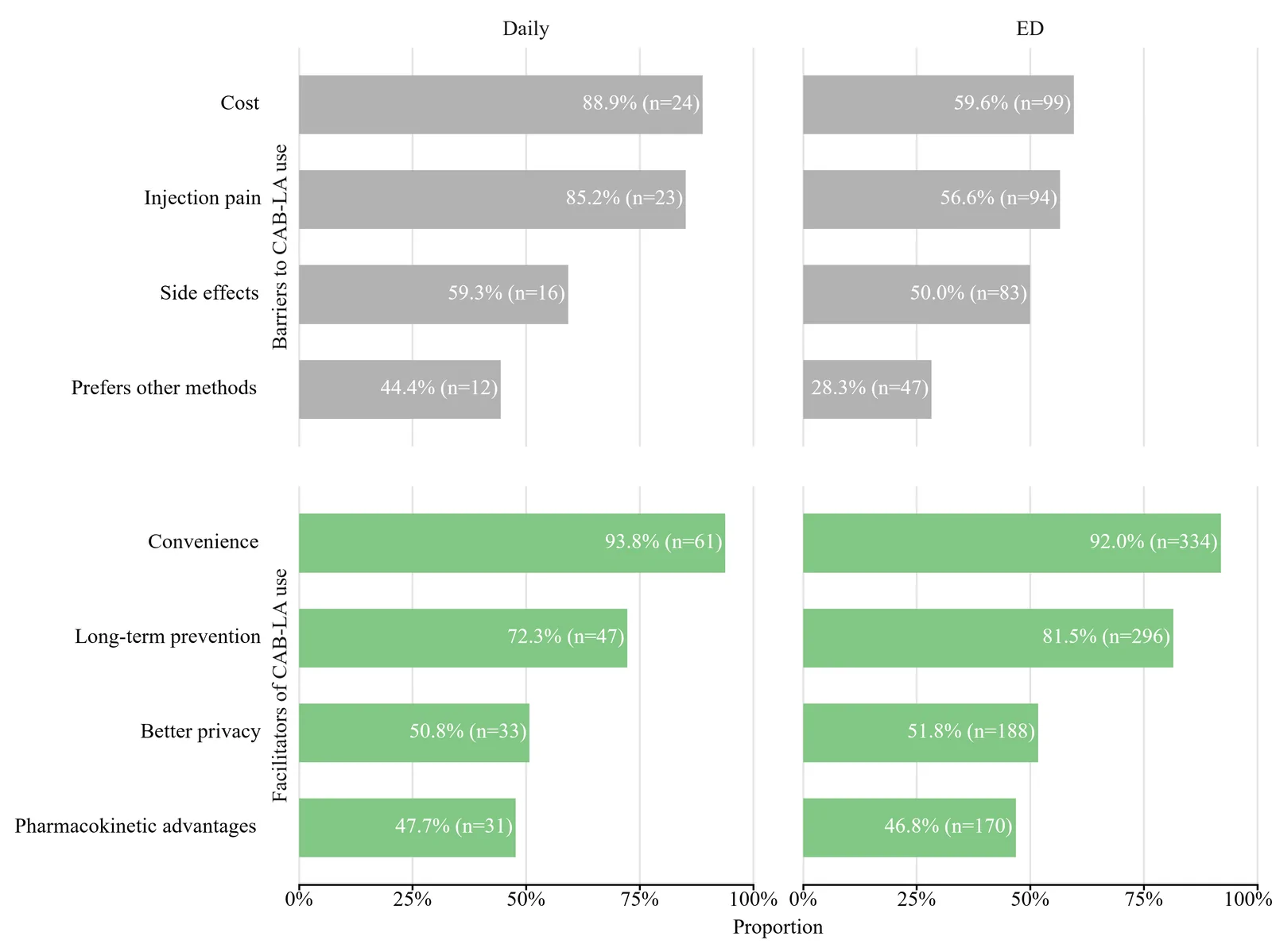
Reported barriers to and facilitators related to hypothetical willingness to use CAB-LA. Facilitators were assessed among participants who expressed hypothetical willingness to use CAB-LA (n=428; daily PrEP: n=65; ED PrEP: n=363), whereas barriers were assessed among participants who did not express willingness (n=193; daily PrEP: n=27; ED PrEP: n=166). Participants could select multiple responses; therefore, the percentages do not sum to 100%. “Convenience,” “Long-term prevention,” “Better privacy,” and “pharmacokinetic advantages” were assessed as separate facilitator options. CAB-LA: long-acting injectable cabotegravir; ED: event-driven; PrEP: pre-exposure prophylaxis.

### Factors Associated With Prior Awareness of CAB-LA

In the multivariable modified Poisson model, event-driven PrEP users had a lower prevalence of prior CAB-LA awareness than daily PrEP users (aPR 0.66, 95% CI 0.50‐0.86; *P*=.003). Participants who reported switching oral PrEP regimens during the past 3 months had a higher prevalence of prior awareness than those who had not switched regimens (aPR 1.34, 95% CI 1.07‐1.68; *P*=.01). No other factors were statistically significantly associated with prior CAB-LA awareness in the adjusted model (Table 2).

**Table 2.** Factors associated with prior awareness of and hypothetical willingness to use long-acting injectable cabotegravir (CAB-LA) among sexually active oral pre-exposure prophylaxis (PrEP) users.^a^

Characteristics	Prior awareness			Hypothetical willingness		
	PR^b^ (95% CI)	aPR^c^ (95% CI)	*P* value	PR (95% CI)	aPR (95% CI)	*P* value
Background information						
Age, per year	1.00 (0.98-1.01)	0.99 (0.97‐1.01)	.42	1.00 (0.99‐1.01)	1.00 (0.99‐1.01)	.90
Ethnicity (other vs Han)	1.36 (0.94‐1.97)	1.41 (0.97‐2.05)	.07	1.05 (0.84‐1.30)	1.11 (0.90‐1.36)	.34
Employment (employed vs student/unemployed)	1.14 (0.85‐1.54)	1.11 (0.79‐1.56)	.55	1.10 (0.94‐1.28)	1.03 (0.86‐1.23)	.73
Education (college or above vs high school or below)	0.82 (0.52‐1.29)	0.88 (0.56‐1.38)	.57	1.02 (0.78‐1.35)	1.06 (0.80‐1.41)	.69
Married/living with a partner (yes vs no)	1.11 (0.82‐1.49)	1.15 (0.83‐1.60)	.39	0.85 (0.70‐1.02)	0.85 (0.70‐1.04)	.12
Monthly income (CNY^d^; >7000 vs ≤7000)	0.99 (0.79‐1.24)	0.93 (0.72‐1.20)	.58	1.11 (0.99‐1.25)	1.08 (0.95‐1.24)	.24
Same-sex sexual behaviors and recent STI^e^ diagnosis						
Chemsex (yes vs no)	0.95 (0.76‐1.19)	0.98 (0.77‐1.24)	.86	1.16 (1.04‐1.30)	1.13 (1.01‐1.26)	.048
Condom use (inconsistent vs consistent)	1.05 (0.84‐1.32)	1.00 (0.79‐1.26)	>.99	1.08 (0.97‐1.20)	1.07 (0.95‐1.19)	.25
Same-sex sexual partners (>1 vs 1)	0.93 (0.71‐1.23)	0.92 (0.70‐1.22)	.56	1.08 (0.93‐1.26)	1.03 (0.88‐1.20)	.71
Receptive anal sex (yes vs no)	0.94 (0.75‐1.17)	0.94 (0.75‐1.18)	.62	1.07 (0.96‐1.20)	1.05 (0.94‐1.17)	.42
Commercial sex (yes vs no)	1.03 (0.70‐1.51)	0.99 (0.66‐1.49)	.97	1.09 (0.91‐1.29)	1.05 (0.88‐1.25)	.61
STI diagnosis in past 3 months (yes vs no)	1.06 (0.75‐1.49)	1.08 (0.76‐1.52)	.67	1.26 (1.11‐1.42)	1.22 (1.08‐1.39)	.002
PrEP-related characteristics						
Oral PrEP regimen (event-driven vs daily)	0.64 (0.50‐0.81)	0.66 (0.50‐0.86)	.003	0.99 (0.85‐1.14)	1.12 (0.95‐1.31)	.17
Oral PrEP medication-instruction knowledge (correct vs incorrect)	1.07 (0.83‐1.37)	1.18 (0.92‐1.52)	.19	0.94 (0.84‐1.06)	0.92 (0.81‐1.03)	.15
PrEP-related stigma score^f^ (per 1-point increase)	0.99 (0.98‐1.01)	1.00 (0.98‐1.01)	.86	0.99 (0.98‐0.99)	0.99 (0.98‐0.99)	.01
Study-defined optimal oral PrEP adherence (met criteria vs did not meet criteria)	1.17 (0.94‐1.46)	1.04 (0.82‐1.32)	.76	1.07 (0.95‐1.19)	1.07 (0.95‐1.20)	.24
Oral PrEP regimen switching (yes vs no)	1.38 (1.11‐1.72)	1.34 (1.07‐1.68)	.01	1.14 (1.02‐1.27)	1.11 (0.99‐1.24)	.07
Prior awareness of CAB-LA (yes vs no)	—^g^	—	—	1.05 (0.94‐1.18)	1.05 (0.93‐1.17)	.44

a Modified Poisson regression with robust SEs was used to estimate prevalence ratios (PRs) and adjusted prevalence ratios (aPRs) with 95% CIs. Primary regression analyses were restricted to participants reporting sexual activity in the past 3 months (n=546) because sexual behavior covariates were structurally inapplicable to participants reporting no recent sexual activity. All candidate covariates were entered simultaneously into the multivariable models irrespective of their statistical significance in univariable analyses. Prior CAB-LA awareness was additionally included in the model for hypothetical willingness.

b PR: prevalence ratio.

c aPR: adjusted prevalence ratio.

d CNY: Chinese yuan; CNY 1=US $0.14 as of January 4, 2024.

e STI: sexually transmitted infection.

f PrEP-related stigma was modeled continuously; the PR and aPR represent the change in prevalence associated with each 1-point increase in stigma score.

g Not applicable.

### Factors Associated With Hypothetical Willingness to Use CAB-LA

In the multivariable model, participants reporting an STI diagnosis in the past 3 months had a higher prevalence of hypothetical willingness to use CAB-LA than those without a recent STI diagnosis (aPR 1.22, 95% CI 1.08‐1.39; *P*=.002). Chemsex was also associated with a modestly higher prevalence of willingness (aPR 1.13, 95% CI 1.01‐1.26; *P*=.048). Higher PrEP-related stigma was associated with a slightly lower prevalence of willingness (aPR 0.99, 95% CI 0.98‐0.99; *P*=.01). No other factors were statistically significantly associated with willingness after adjustment (Table 2).

### Sensitivity Analyses

Sensitivity analyses generally supported the primary findings. Associations of event-driven PrEP use and regimen switching with prior CAB-LA awareness remained consistent after excluding adherence and in the full-sample analysis (Table S1 in Multimedia Appendix 2). For hypothetical willingness, the associations with recent STI diagnosis, chemsex, and PrEP-related stigma were similar after excluding adherence or prior CAB-LA awareness. In the full-sample analysis, recent STI diagnosis and PrEP-related stigma remained associated with hypothetical willingness, and regimen switching was also associated with higher willingness (aPR 1.12, 95% CI 1.01‐1.25; *P*=.03; Table S2 in Multimedia Appendix 2). No evidence of problematic multicollinearity was observed, with all VIF values below 1.5 in the primary and sensitivity models.

## Discussion

### Principal Findings

In this web-based cross-sectional study of oral PrEP users in China obtaining PrEP online, we found a marked contrast between prior awareness of CAB-LA and hypothetical willingness to consider it. Although only about one-third of participants had previously heard of CAB-LA, more than two-thirds expressed hypothetical willingness to use it after receiving standardized information, suggesting that limited familiarity with this prevention option may coexist with substantial interest when CAB-LA is presented as an alternative PrEP modality. Differences in awareness by oral PrEP regimen and recent regimen switching, together with associations of willingness with recent STI diagnosis, chemsex, and PrEP-related stigma, further suggest that information needs and preferences for long-acting PrEP may vary across users. The strong appeal of convenience, alongside concerns about cost, injection-related pain, and adverse effects, indicates that the future introduction and delivery of CAB-LA will depend not only on increasing awareness but also on whether it can be provided in an affordable and acceptable way.

Prior awareness of CAB-LA remained limited despite participants already having experience with oral PrEP. The 34.8% awareness observed in our study was similar to the 35.8% reported among former participants of the oral PrEP demonstration project (China Real-World Oral Intake of PrEP [CROPrEP] study) in China [17] but lower than the 51.9% reported among oral PrEP users in the United States [22] and 64% in the French ANRS-PREVENIR cohort [18]. Although differences in populations and settings limit direct comparison, these findings suggest relatively limited awareness of long-acting injectable PrEP among Chinese oral PrEP users. Awareness was particularly low among event-driven PrEP users. This may partly reflect differences in PrEP use and service engagement, as event-driven PrEP is more commonly preferred by users with less frequent or more predictable sexual activity [23]. Among Chinese MSM obtaining event-driven PrEP online, only 21.3% reported PrEP-related follow-up in the preceding 3 months despite most having previously received PrEP counseling [24], suggesting fewer opportunities for continued exposure to updated PrEP information. Although information sources and counseling exposure were not directly assessed in our study, online PrEP platforms may provide an important channel for delivering updated information about oral and long-acting PrEP options and supporting informed choice [7,24,25].

Recent switching between daily and event-driven oral PrEP use was also associated with greater prior CAB-LA awareness. PrEP regimen switching often occurs in response to changing sexual activity, lifestyle, or prevention preferences and may involve active reassessment of users’ PrEP needs [26]. Users reconsidering their oral PrEP regimen may therefore be more likely to seek or encounter information about alternative prevention options. However, because the timing of CAB-LA awareness relative to regimen switching was not assessed, reverse causality is also possible; prior exposure to information about CAB-LA may have prompted broader reconsideration of available PrEP strategies. Thus, regimen switching should not be interpreted as leading to greater CAB-LA awareness, and both may instead reflect greater engagement in PrEP decision-making. Nevertheless, discussions around regimen choice or switching may provide useful opportunities to introduce balanced information about available PrEP options.

Despite limited prior awareness, 68.9% of participants expressed hypothetical willingness to use CAB-LA after receiving standardized information. This proportion was somewhat lower than the 77.8% willingness to use free long-acting injectable (LAI)–PrEP reported among former CROPrEP participants. Notably, only 40.8% of CROPrEP participants were willing to pay for LAI-PrEP, suggesting that the stated willingness may be sensitive to payment conditions [17]. In our study, convenience and long-term prevention were the most frequently reported facilitators, consistent with previous studies identifying reduced pill burden as an important appeal of injectable PrEP [15,27]. Cost is particularly important in interpreting these responses. At the time of data collection, CAB-LA had not yet been commercially available in China, and the annual cost of approximately CNY 50,000 presented to participants was a standardized estimate rather than an observed Chinese market price. This amount was substantially higher than participants’ current oral PrEP expenditure, which had a median of CNY 300 (IQR 200-300) per month. PrEP for HIV prevention in China is generally paid out of pocket rather than routinely reimbursed through national basic medical insurance, making users’ willingness and ability to pay particularly relevant to eventual uptake [28]. A previous Chinese study similarly found that 58.2% were concerned about CAB-LA cost and only 14.7% were willing to pay more than CNY 1200 every 2 months [14]. Together, the high hypothetical willingness observed in our study should not be interpreted as expected uptake. Actual use is likely to depend on the market price and affordability of CAB-LA and whether future reimbursement, subsidies, or other financing mechanisms reduce the out-of-pocket burden, in addition to injection acceptability and related service requirements.

Recent STI diagnosis was independently associated with greater hypothetical willingness to use CAB-LA, consistent with previous studies [17,29]. This association may indicate that individuals with more immediate sexual health or prevention needs are more open to considering alternative PrEP options, although the underlying pathway cannot be determined from cross-sectional data. Chemsex was also associated with greater willingness, although the association in our study was modest. Similar findings have been reported among former CROPrEP participants who used recreational drugs and among current oral PrEP users in the Netherlands, where chemsex was independently associated with intention to use LAI-PrEP [17,30,31]. Because oral PrEP adherence itself was not associated with willingness in our adjusted analysis, the association with chemsex should not be interpreted as evidence that poorer oral PrEP adherence explains greater interest in CAB-LA. Instead, long-acting PrEP may appeal to some users whose sexual contexts or prevention needs vary over time. Finally, greater PrEP-related stigma was associated with a slightly lower prevalence of hypothetical willingness, consistent with previous evidence linking anticipated PrEP-related disapproval with lower willingness to use LAI-PrEP [32,33]. Because our measure assessed general PrEP-related stigma rather than CAB-LA–specific stigma, this association may reflect broader social concerns surrounding PrEP rather than attitudes specific to injectable prevention.

These findings suggest that online PrEP platforms may have a role beyond facilitating access to oral PrEP by also supporting informed consideration of emerging prevention options. Digital PrEP services could incorporate balanced and regularly updated information on different PrEP modalities and provide opportunities for counseling as users consider their PrEP options [8]. Information about new options should address not only potential advantages, such as convenience and reduced dosing burden, but also practical concerns that may shape users’ decisions, including cost, injection-related discomfort, adverse effects, and follow-up requirements [25]. In this way, online PrEP platforms may serve as an additional channel for supporting informed PrEP choice as the range of prevention options expands.

### Limitations

This study has several limitations. First, participants were recruited from a single online PrEP platform and represented a highly educated and relatively higher-income population. Although high educational attainment has also been commonly reported in previous PrEP cohorts and intervention studies in China [3,34], the proportion with college-level education or above was relatively high in our sample. The findings should therefore be generalized cautiously to PrEP users who obtain medication through clinics, community-based services, informal channels, or other online platforms, as well as to populations with lower educational and socioeconomic status. This socioeconomic selection is particularly relevant to the affordability findings. Although high cost was reported by 63.7% of participants who did not express willingness, affordability concerns may be greater among lower-income and more socioeconomically diverse populations, and our sample may therefore underestimate the importance of cost as a barrier to CAB-LA uptake. Second, sexual orientation was not directly assessed, limiting interpretation across sexual orientation groups. Third, willingness was hypothetical and was assessed immediately after participants received a standardized description of CAB-LA, including its potential benefits, risks, and estimated annual cost. Responses may therefore have been influenced by how this information was presented and may differ from decisions made after full clinical counseling or under actual pricing, availability, and service conditions; they should not be interpreted as future uptake. Fourth, the cross-sectional design precludes establishing temporal or causal relationships. In particular, the direction of the association between regimen switching and CAB-LA awareness cannot be determined. Fifth, sexual behaviors, chemsex, and recent STI diagnoses were self-reported and may be affected by recall or social desirability bias. Recent STI diagnosis reflected self-reported clinical diagnoses rather than laboratory-confirmed incidence and may therefore have missed asymptomatic or undiagnosed infections. Finally, PrEP-related stigma was assessed using a general PrEP stigma measure rather than a CAB-LA–specific measure. Although the Chinese-language version underwent translation, back-translation, and pilot testing, a separate formal psychometric validation was not conducted in this study.

### Conclusions

Among oral PrEP users in China obtaining PrEP online, prior awareness of CAB-LA was limited, whereas hypothetical willingness to consider it was common after standardized information was provided. Prior awareness differed by oral PrEP regimen and recent regimen switching, whereas hypothetical willingness was associated with recent STI diagnosis, chemsex, and PrEP-related stigma. Convenience was a major appeal, while cost, injection-related discomfort, and concerns about adverse effects may remain important considerations for future CAB-LA use. These findings support improving access to balanced information on PrEP options and addressing practical barriers as long-acting PrEP becomes more widely available.

## Supplementary material

10.2196/96586 Multimedia Appendix 1 Standardized information about long-acting injectable cabotegravir (CAB-LA) provided before the hypothetical willingness assessment.

10.2196/96586 Multimedia Appendix 2 Sensitivity analyses of factors associated with prior long-acting injectable cabotegravir (CAB-LA) awareness and hypothetical willingness to use CAB-LA.

10.2196/96586 Checklist 1 CHERRIES checklist.

## Abbreviations

aPR: adjusted prevalence ratio
CAB-LA: long-acting injectable cabotegravir
CDC: Center for Disease Control and Prevention
CHERRIES: Checklist for Reporting Results of Internet E-Surveys
CROPrEP: China Real-World Oral Intake of PrEP
LAI: long-acting injectable
MSM: men who have sex with men
PR: prevalence ratio
PrEP: pre-exposure prophylaxis
RECOPE: Real-world E-consumer Cohort of Oral PrEP
STI: sexually transmitted infection
VIF: variance inflation factor
