# Supplementary material for: Awareness of and Hypothetical Willingness to Use Long-Acting Injectable Cabotegravir and Associated Factors Among Oral Pre-Exposure Prophylaxis Users Who Obtained the Medicine Online in China: Web-Based Cross-Sectional Study
Source: J Med Internet Res. 2026 Oct 28;28:e96586. doi: 10.2196/96586 (PMC13716419; doi:10.2196/96586)
Supplement: Supplementary file 1 [file jmir-v28-e96586-s001.docx]

Multimedia Appendix 1. Standardized Information About CAB-LA Provided Before the Hypothetical Willingness Assessment

*Document note. The Chinese text below reproduces the original participant-facing wording used in the survey. The English text is provided solely as a translation for publication and was not shown to participants.*

# Original participant-facing information (Chinese)

长效注射用卡博特韦（long-acting injectable cabotegravir，CAB-LA）是一种用于HIV暴露前预防（PrEP）的注射药物。CAB-LA采用肌内注射方式给药，前两次注射间隔4周，此后每8周注射一次。临床研究显示，CAB-LA在预防HIV感染方面具有较高的保护效果。常见不良反应包括注射部位反应，如疼痛、肿胀或其他注射部位不适。在开始使用CAB-LA前以及使用期间需要定期进行HIV检测。如果在未被发现的HIV感染情况下使用CAB-LA，可能增加产生整合酶抑制剂耐药的风险。因此，在CAB-LA使用期间需要按照要求进行HIV检测，并按时接受注射。在本次调查开展时，CAB-LA的估计年费用约为人民币50,000元。

随后，参与者回答以下问题：

**“如果CAB-LA今后可在中国用于HIV预防，您是否愿意使用CAB-LA？”**

回答采用5级量表：

1 = 非常不愿意；2 = 不愿意；3 = 不确定；4 = 愿意；5 = 非常愿意

# English translation for publication

Long-acting injectable cabotegravir (CAB-LA) is an injectable medication for HIV pre-exposure prophylaxis. CAB-LA is administered by intramuscular injection. The first 2 injections are given 4 weeks apart, followed by an injection every 8 weeks. Clinical studies have shown that CAB-LA is highly effective in preventing HIV infection. Common adverse effects include injection-site reactions, such as pain, swelling, or other discomfort after injection. HIV testing is required before and during CAB-LA use. If CAB-LA is administered to a person with undiagnosed HIV infection, there is a risk of developing resistance to integrase inhibitors. Regular HIV testing and timely injections are therefore required during CAB-LA use. At the time of this survey, the estimated annual cost of CAB-LA was approximately CNY 50,000.

Participants were then asked:

**“If CAB-LA becomes available for HIV prevention in China, would you be willing to use it?”**

Responses were recorded on a 5-point scale:

1 = Definitely unwilling; 2 = Unwilling; 3 = Unsure; 4 = Willing; 5 = Strongly willing

# Contextual note on the annual cost estimate

The approximately CNY 50,000 annual cost was part of the original standardized information shown to participants. When the questionnaire was developed, there was no observed Chinese market price for CAB-LA for HIV prevention. The value was used as a pragmatic survey estimate informed by pricing context available at the time, including PrEP drug-cost assumptions reported in Chinese cost-effectiveness literature and contemporaneous European pricing of long-acting cabotegravir. It was not intended to represent an observed or official Chinese market price.

## References for the contextual note

Mi Y, Zeng Y, Wang P, Zhou M, Cheng F. Cost-Effectiveness of Pre-exposure Prophylaxis Among Men Who Have Sex With Men in China: A Systematic Review. Front Public Health. 2022;10:809268. doi:10.3389/fpubh.2022.809268.

Journal officiel de la République française. Avis relatif aux prix de spécialités pharmaceutiques. December 17, 2021. VOCABRIA 600 mg prolonged-release injectable suspension: public price €900.79 per vial.

European Medicines Agency. Apretude (cabotegravir): European Public Assessment Report. EU marketing authorization issued September 15, 2023.

Toby Pepperrell, Samuel Cross, Andrew Hill, Cabotegravir—Global Access to Long-Acting Pre-exposure Prophylaxis for HIV, *Open Forum Infectious Diseases*, Volume 10, Issue 1, January 2023, ofac673, <https://doi.org/10.1093/ofid/ofac673>
