# Supplementary material for: Awareness of and Hypothetical Willingness to Use Long-Acting Injectable Cabotegravir and Associated Factors Among Oral Pre-Exposure Prophylaxis Users Who Obtained the Medicine Online in China: Web-Based Cross-Sectional Study
Source: J Med Internet Res. 2026 Oct 28;28:e96586. doi: 10.2196/96586 (PMC13716419; doi:10.2196/96586)
Supplement: Supplementary file 2 [file jmir-v28-e96586-s002.docx]

Table S1. Sensitivity analyses of factors associated with prior awareness of CAB-LA

| **Characteristic** | **Primary model**  **(n=546)** | **Excluding adherence**  **(n=546)** | **Full sample**  **(N=621)** |
| --- | --- | --- | --- |
| Age, per year | 0.99 (0.97–1.01); P=.420 | 0.99 (0.97–1.01); P=.427 | 0.99 (0.97–1.01); P=.381 |
| Ethnicity, Other vs Han | 1.41 (0.97–2.05); P=.070 | 1.41 (0.97–2.05); P=.070 | 1.33 (0.92–1.92); P=.130 |
| Employment, employed vs student/unemployed | 1.11 (0.79–1.56); P=.553 | 1.11 (0.79–1.56); P=.549 | 1.10 (0.80–1.51); P=.575 |
| Education, college or above vs high school or below | 0.88 (0.56–1.38); P=.570 | 0.88 (0.56–1.38); P=.572 | 0.99 (0.64–1.54); P=.962 |
| Married/living with a partner, yes vs no | 1.15 (0.83–1.60); P=.390 | 1.15 (0.83–1.59); P=.399 | 1.20 (0.88–1.64); P=.245 |
| Monthly income, >7000 vs ≤7000 CNY | 0.93 (0.72–1.20); P=.582 | 0.93 (0.72–1.20); P=.565 | 0.90 (0.71–1.15); P=.396 |
| STI diagnosis in past 3 months, yes vs no | 1.08 (0.76–1.52); P=.672 | 1.08 (0.76–1.52); P=.677 | 1.10 (0.79–1.55); P=.562 |
| Chemsex, yes vs no | 0.98 (0.77–1.24); P=.856 | 0.98 (0.77–1.24); P=.836 | — |
| Condom use, inconsistent vs consistent | 1.00 (0.79–1.26); P=.999 | 1.00 (0.79–1.26); P=.989 | — |
| Same-sex sexual partners, >1 vs 1 | 0.92 (0.70–1.22); P=.560 | 0.92 (0.69–1.21); P=.536 | — |
| Receptive anal sex, yes vs no | 0.94 (0.75–1.18); P=.621 | 0.95 (0.76–1.19); P=.642 | — |
| Commercial sex, yes vs no | 0.99 (0.66–1.49); P=.974 | 1.00 (0.66–1.50); P=.986 | — |
| Oral PrEP regimen, ED vs daily | 0.66 (0.50–0.86); P=.003 | 0.64 (0.50–0.83); P=.001 | 0.64 (0.50–0.83); P=.001 |
| Oral PrEP medication-instruction knowledge, correct vs incorrect | 1.18 (0.92–1.52); P=.190 | 1.19 (0.92–1.53); P=.182 | 1.20 (0.94–1.53); P=.149 |
| PrEP-related stigma score, per 1-point increase | 1.00 (0.98–1.01); P=.864 | 1.00 (0.98–1.01); P=.831 | 0.99 (0.98–1.01); P=.430 |
| Study-defined optimal oral PrEP adherence,  met criteria vs did not meet criteria | 1.04 (0.82–1.32); P=.761 | — | — |
| Oral PrEP regimen switching, yes vs no | 1.34 (1.07–1.68); P=.011 | 1.34 (1.07–1.68); P=.011 | 1.27 (1.02–1.57); P=.031 |
| Sexual activity in past 3 months, yes vs no | — | — | 1.48 (0.95–2.30); P=.083 |

**Note:** Values are adjusted prevalence ratios (aPRs) with 95% CIs estimated using modified Poisson regression with robust standard errors. The primary model was restricted to participants reporting sexual activity in the past 3 months (n=546). The adherence sensitivity model excluded oral PrEP adherence because adherence was assessed using regimen-specific criteria for daily and event-driven PrEP. The full-sample model included all 621 participants and excluded sexual-behavior variables and regimen-specific adherence that were structurally inapplicable to participants reporting no recent sexual activity; recent sexual activity was included as a covariate. All remaining covariates were entered simultaneously into each model. An em dash indicates that the variable was not included in the corresponding model. CAB-LA indicates long-acting injectable cabotegravir; CNY, Chinese yuan; ED, event-driven; PrEP, pre-exposure prophylaxis; STI, sexually transmitted infection.

Table S2. Sensitivity analyses of factors associated with hypothetical willingness to use CAB-LA

| **Characteristic** | **Primary model**  **(n=546)** | **Excluding adherence**  **(n=546)** | **Excluding prior awareness**  **(n=546)** | **Full sample**  **(N=621)** |
| --- | --- | --- | --- | --- |
| Age, per year | 1.00 (0.99–1.01); P=.899 | 1.00 (0.99–1.01); P=.956 | 1.00 (0.99–1.01); P=.884 | 1.00 (0.99–1.01); P=.849 |
| Ethnicity  (Other vs Han) | 1.11 (0.90–1.36); P=.339 | 1.11 (0.90–1.36); P=.344 | 1.11 (0.90–1.37); P=.313 | 1.04 (0.85–1.26); P=.708 |
| Employment  (employed vs student/unemployed) | 1.03 (0.86–1.23); P=.731 | 1.03 (0.86–1.23); P=.722 | 1.03 (0.86–1.24); P=.720 | 1.08 (0.91–1.27); P=.377 |
| Education  (college or above vs high school or below) | 1.06 (0.80–1.41); P=.688 | 1.06 (0.80–1.41); P=.686 | 1.06 (0.79–1.40); P=.708 | 1.00 (0.79–1.27); P=.997 |
| Married/living with a partner  (yes vs no) | 0.85 (0.70–1.04); P=.107 | 0.85 (0.70–1.03); P=.097 | 0.85 (0.70–1.04); P=.113 | 0.88 (0.73–1.05); P=.149 |
| Monthly income  (>7000 vs ≤7000 CNY) | 1.08 (0.95–1.24); P=.245 | 1.08 (0.94–1.23); P=.275 | 1.08 (0.95–1.24); P=.251 | 1.06 (0.94–1.20); P=.363 |
| STI diagnosis in past 3 months  (yes vs no) | 1.22 (1.08–1.39); P=.002 | 1.22 (1.07–1.38); P=.002 | 1.22 (1.08–1.39); P=.002 | 1.23 (1.08–1.39); P=.001 |
| Chemsex  (yes vs no) | 1.13 (1.01–1.26); P=.048 | 1.13 (1.01–1.26); P=.048 | 1.13 (1.00–1.25); P=.049 | — |
| Condom use  (inconsistent vs consistent) | 1.07 (0.95–1.19); P=.253 | 1.07 (0.96–1.19); P=.241 | 1.07 (0.95–1.19); P=.254 | — |
| Same-sex sexual partners  (>1 vs 1) | 1.03 (0.88–1.20); P=.714 | 1.02 (0.87–1.19); P=.812 | 1.03 (0.88–1.20); P=.729 | — |
| Receptive anal sex  (yes vs no) | 1.05 (0.94–1.17); P=.416 | 1.05 (0.94–1.18); P=.345 | 1.05 (0.94–1.17); P=.424 | — |
| Commercial sex  (yes vs no) | 1.05 (0.88–1.25); P=.609 | 1.06 (0.89–1.26); P=.547 | 1.05 (0.88–1.25); P=.611 | — |
| Oral PrEP regimen  (ED vs daily) | 1.12 (0.95–1.31); P=.172 | 1.09 (0.93–1.27); P=.300 | 1.11 (0.95–1.30); P=.202 | 1.08 (0.93–1.25); P=.313 |
| Oral PrEP medication-instruction knowledge  (correct vs incorrect) | 0.92 (0.81–1.03); P=.146 | 0.92 (0.82–1.04); P=.167 | 0.92 (0.82–1.03); P=.159 | 0.93 (0.83–1.03); P=.174 |
| PrEP-related stigma score  (per 1-point increase) | 0.99 (0.98–1.00); P=.014 | 0.99 (0.98–1.00); P=.008 | 0.99 (0.98–1.00); P=.014 | 0.99 (0.98–0.99); P<.001 |
| Study-defined optimal oral PrEP adherence  (met criteria vs did not meet criteria) | 1.07 (0.95–1.20); P=.245 | — | 1.07 (0.95–1.20); P=.244 | — |
| Oral PrEP regimen switching  (yes vs no) | 1.11 (0.99–1.24); P=.072 | 1.11 (0.99–1.23); P=.077 | 1.11 (1.00–1.24); P=.056 | 1.12 (1.01–1.25); P=.027 |
| Prior awareness of CAB-LA  (yes vs no) | 1.05 (0.93–1.17); P=.437 | 1.05 (0.94–1.17); P=.434 | — | 1.05 (0.95–1.17); P=.331 |
| Sexual activity in past 3 months  (yes vs no) | — | — | — | 1.04 (0.87–1.24); P=.642 |

Note: Values are adjusted prevalence ratios (aPRs) with 95% CIs estimated using modified Poisson regression with robust standard errors. The primary model was restricted to participants reporting sexual activity in the past 3 months (n=546). The adherence sensitivity model excluded oral PrEP adherence because adherence was assessed using regimen-specific criteria for daily and event-driven PrEP. The full-sample model included all 621 participants and excluded sexual-behavior variables and regimen-specific adherence that were structurally inapplicable to participants reporting no recent sexual activity; recent sexual activity was included as a covariate. All remaining covariates were entered simultaneously into each model. An em dash indicates that the variable was not included in the corresponding model. CAB-LA indicates long-acting injectable cabotegravir; CNY, Chinese yuan; ED, event-driven; PrEP, pre-exposure prophylaxis; STI, sexually transmitted infection.
