# Supplementary material for: Awareness of and Hypothetical Willingness to Use Long-Acting Injectable Cabotegravir and Associated Factors Among Oral Pre-Exposure Prophylaxis Users Who Obtained the Medicine Online in China: Web-Based Cross-Sectional Study
Source: J Med Internet Res. 2026 Oct 28;28:e96586. doi: 10.2196/96586 (PMC13716419; doi:10.2196/96586)
Supplement: Supplementary file 3 [file jmir-v28-e96586-s003.docx]

**Checklist for Reporting Results of Internet E-Surveys (CHERRIES)**

| ***Checklist Item*** | ***Explanation*** | ***Page number / response*** |
| --- | --- | --- |
| Describe survey design | Describe target population, sample frame. Is the sample a convenience sample? (In “open” surveys this is most likely.) | pp 5-6. This was a cross-sectional analysis of baseline data from RECOPE. The target population was real-world online oral PrEP users in China. All HeHealth users who had purchased oral PrEP in the preceding 1-3 months were invited during the recruitment period. The analytic sample was a platform-based convenience sample from one online PrEP service. |
| IRB approval | Mention whether the study has been approved by an IRB. | p 9. The study was approved by the Institutional Review Board of Tsinghua University (20230195). |
| Informed consent | Describe the informed consent process. Where were the participants told the length of time of the survey, which data were stored and where and for how long, who the investigator was, and the purpose of the study? | pp 6, 8-9. Electronic informed consent was obtained through WeChat before enrollment. The consent form identified the research team and study purpose and described the procedures, expected survey time (approximately 5-10 minutes), risks and benefits, confidentiality, voluntary participation, and where study data would be stored and for how long. Participants clicked an agreement button before proceeding. |
| Data protection | If any personal information was collected or stored, describe what mechanisms were used to protect unauthorized access. | p 9. The questionnaire dataset used study IDs rather than names or other direct identifiers. Individual responses were accessible only to the research team and were not available to HeHealth. Electronic consent was used instead of handwritten signatures to reduce collection of identifying information. |
| Development and testing | State how the survey was developed, including whether the usability and technical functionality of the electronic questionnaire had been tested before fielding the questionnaire. | pp 8, 10. The complete formal Wenjuanxing electronic questionnaire was pilot-tested by 8 oral PrEP users before data collection; unclear wording was revised based on their feedback. Regimen-specific PrEP knowledge items were reviewed by HIV prevention and PrEP experts, and the Chinese HPSS underwent translation, back-translation, and pilot testing. |
| Open survey versus closed survey | An “open survey” is a survey open for each visitor of a site, while a closed survey is only open to a sample which the investigator knows (password-protected survey). | p 8. Closed survey. It was not publicly accessible. Each enrolled participant received a unique study ID that served as the access passcode. |
| Contact mode | Indicate whether or not the initial contact with the potential participants was made on the Internet. (Investigators may also send out questionnaires by mail and allow for Web-based data entry.) | p 6. Initial contact was made online through HeHealth's internal messaging system. Interested users were referred to the research team through WeChat. |
| Advertising the survey | How/where was the survey announced or advertised? Some examples are offline media (newspapers), or online (mailing lists – If yes, which ones?) or banner ads (Where were these banner ads posted and what did they look like?). It is important to know the wording of the announcement as it will heavily influence who chooses to participate. Ideally the survey announcement should be published as an appendix. | p 6. The survey was not publicly advertised. HeHealth sent an internal study invitation to all platform users who had purchased oral PrEP in the preceding 1-3 months during recruitment. |
| Web/E-mail | State the type of e-survey (eg, one posted on a Web site, or one sent out through e-mail). If it is an e-mail survey, were the responses entered manually into a database, or was there an automatic method for capturing responses? | p 8. This was a self-administered web-based questionnaire hosted on Wenjuanxing (www.wjx.com). Responses were captured electronically in the survey database. |
| Context | Describe the Web site (for mailing list/newsgroup) in which the survey was posted. What is the Web site about, who is visiting it, what are visitors normally looking for? Discuss to what degree the content of the Web site could pre-select the sample or influence the results. For example, a survey about vaccination on a anti-immunization Web site will have different results from a Web survey conducted on a government Web site | pp 5-6, 26. HeHealth is an internet-based PrEP service and distribution platform used by people seeking online PrEP access. Recruitment through one PrEP-specific platform preselected recent oral PrEP purchasers and may limit generalizability to clinic-based, community-based, informal-channel, and other online PrEP users. |
| Mandatory/voluntary | Was it a mandatory survey to be filled in by every visitor who wanted to enter the Web site, or was it a voluntary survey? | p 9. Participation was voluntary. The survey was not required to access HeHealth services. |
| Incentives | Were any incentives offered (eg, monetary, prizes, or non-monetary incentives such as an offer to provide the survey results)? | p 9. Participants received a HeHealth voucher valued at CNY 50 (approximately US $7) after each valid completed questionnaire. |
| Time/Date | In what timeframe were the data collected? | p 5. Baseline data were collected from December 6, 2023, to January 4, 2024. |
| Randomization of items or questionnaires | To prevent biases items can be randomized or alternated. | Not used. Questionnaire items and response options were presented in a fixed order; no item or questionnaire randomization was applied. |
| Adaptive questioning | Use adaptive questioning (certain items, or only conditionally displayed based on responses to other items) to reduce number and complexity of the questions. | p 14. Yes. Prespecified automatic skip logic displayed conditional questions only when applicable, including sexual-behavior measures conditional on recent sexual activity. |
| Number of Items | What was the number of questionnaire items per page? The number of items is an important factor for the completion rate. | Approximately 30 items were displayed per page. The exact number varied because of automatic skip logic and structural applicability. |
| Number of screens (pages) | Over how many pages was the questionnaire distributed? The number of items is an important factor for the completion rate. | The questionnaire was distributed over 3 electronic pages. |
| Completeness check | It is technically possible to do consistency or completeness checks before the questionnaire is submitted. Was this done, and if “yes”, how (usually JAVAScript)? An alternative is to check for completeness after the questionnaire has been submitted (and highlight mandatory items). If this has been done, it should be reported. All items should provide a non-response option such as “not applicable” or “rather not say”, and selection of one response option should be enforced. | pp 8, 14. All applicable items were mandatory, and Wenjuanxing required a response before the participant could proceed or submit. Conditional items were handled by automatic skip logic. Three attention-check items were included; at least 2 correct responses were required for validity. |
| Review step | State whether respondents were able to review and change their answers (eg, through a Back button or a Review step which displays a summary of the responses and asks the respondents if they are correct). | Yes. Before final submission, participants could use the Back function to review and change answers on earlier pages. |
| Unique site visitor | If you provide view rates or participation rates, you need to define how you determined a unique visitor. There are different techniques available, based on IP addresses or cookies or both. | p 8. Unique participants were identified by study ID. Each ID allowed only one submission. No public-site visitor count was used. |
| View rate (Ratio of unique survey visitors/unique site visitors) | Requires counting unique visitors to the first page of the survey, divided by the number of unique site visitors (not page views!). It is not unusual to have view rates of less than 0.1 % if the survey is voluntary. | Not applicable. The questionnaire was a closed, ID-restricted survey rather than a survey displayed to general site visitors, so a CHERRIES view-rate denominator was not available. |
| Participation rate (Ratio of unique visitors who agreed to participate/unique first survey page visitors) | Count the unique number of people who filled in the first survey page (or agreed to participate, for example by checking a checkbox), divided by visitors who visit the first page of the survey (or the informed consents page, if present). This can also be called “recruitment” rate. | Not calculated using the CHERRIES first-page-visitor formula because access occurred only after eligibility screening and electronic consent. In total, 680 eligible participants were issued questionnaire access (pp 6-7). |
| Completion rate (Ratio of users who finished the survey/users who agreed to participate) | The number of people submitting the last questionnaire page, divided by the number of people who agreed to participate (or submitted the first survey page). This is only relevant if there is a separate “informed consent” page or if the survey goes over several pages. This is a measure for attrition. Note that “completion” can involve leaving questionnaire items blank. This is not a measure for how completely questionnaires were filled in. (If you need a measure for this, use the word “completeness rate”.) | pp 6-7. The completion rate was 96.6% (657/680). The 23 noncompleters did not submit any questionnaire and had no study-ID access record; no partially completed questionnaires were recorded. |
| Cookies used | Indicate whether cookies were used to assign a unique user identifier to each client computer. If so, mention the page on which the cookie was set and read, and how long the cookie was valid. Were duplicate entries avoided by preventing users access to the survey twice; or were duplicate database entries having the same user ID eliminated before analysis? In the latter case, which entries were kept for analysis (eg, the first entry or the most recent)? | p 8. Cookies were not used for unique identification or duplicate control. Duplicate submissions were prevented through unique study-ID access, with one permitted submission per ID. |
| IP check | Indicate whether the IP address of the client computer was used to identify potential duplicate entries from the same user. If so, mention the period of time for which no two entries from the same IP address were allowed (eg, 24 hours). Were duplicate entries avoided by preventing users with the same IP address access to the survey twice; or were duplicate database entries having the same IP address within a given period of time eliminated before analysis? If the latter, which entries were kept for analysis (eg, the first entry or the most recent)? | p 8. IP-address checks were not used, and no device-based restriction was applied. |
| Log file analysis | Indicate whether other techniques to analyze the log file for identification of multiple entries were used. If so, please describe. | p 8. No separate log-file analysis was used to identify duplicate entries because access and submission were controlled by unique study IDs. ID access records were checked to establish that the 23 noncompleters had not accessed the questionnaire. |
| Registration | In “closed” (non-open) surveys, users need to login first and it is easier to prevent duplicate entries from the same user. Describe how this was done. For example, was the survey never displayed a second time once the user had filled it in, or was the username stored together with the survey results and later eliminated? If the latter, which entries were kept for analysis (eg, the first entry or the most recent)? | p 8. After enrollment, each participant entered a unique study ID as the Wenjuanxing passcode. The survey was not displayed publicly, and each ID could submit only once. The questionnaire dataset retained the study ID rather than a platform username or direct identifier. |
| Handling of incomplete questionnaires | Were only completed questionnaires analyzed? Were questionnaires which terminated early (where, for example, users did not go through all questionnaire pages) also analyzed? | pp 6-7, 14. Only submitted, valid baseline questionnaires were analyzed. Of 680 participants issued access, 657 submitted the questionnaire. The other 23 made no submission and had no ID access record; there were no early-terminated or partially submitted questionnaires. Conditional nonavailability resulted from skip patterns or structural inapplicability, not item nonresponse. |
| Questionnaires submitted with an atypical timestamp | Some investigators may measure the time people needed to fill in a questionnaire and exclude questionnaires that were submitted too soon. Specify the timeframe that was used as a cut-off point, and describe how this point was determined. | p 8. No minimum or maximum completion-time cutoff was used. Response validity was assessed using 3 attention-check items, with at least 2 correct answers required. |
| Statistical correction | Indicate whether any methods such as weighting of items or propensity scores have been used to adjust for the non-representative sample; if so, please describe the methods. | pp 13-14. No survey weighting, propensity-score adjustment, or other statistical correction for sample representativeness was applied. Modified Poisson regression with robust standard errors was used for association analyses, and sensitivity analyses assessed specified modeling choices. |

This checklist has been modified from Eysenbach G. Improving the quality of Web surveys: the Checklist for Reporting Results of Internet E-Surveys (CHERRIES). J Med Internet Res. 2004 Sep 29;6(3):e34 [erratum in J Med Internet Res. 2012; 14(1): e8.]. Article available at [https://www.jmir.org/2004/3/e34](https://www.jmir.org/2004/3/e34/)/; erratum available <https://www.jmir.org/2012/1/e8/>. Copyright ©Gunther Eysenbach. Originally published in the [Journal of Medical Internet](http://www.jmir.org) Research, 29.9.2004 and 04.01.2012.

This is an open-access article distributed under the terms of the Creative Commons Attribution License (<https://creativecommons.org/licenses/by/2.0/>), which permits unrestricted use, distribution, and reproduction in any medium, provided the original work, first published in the Journal of Medical Internet Research, is properly cited.
